# Functional Evolution in the Plant *SQUAMOSA-PROMOTER BINDING PROTEIN-LIKE* (*SPL*) Gene Family

**DOI:** 10.3389/fpls.2013.00080

**Published:** 2013-04-05

**Authors:** Jill C. Preston, Lena C. Hileman

**Affiliations:** ^1^Plant Biology, The University of VermontBurlington, VT, USA; ^2^Ecology and Evolutionary Biology, The University of KansasLawrence, KS, USA

**Keywords:** *SPL* genes, gene duplication, phase change, flowering time, branching architecture, developmental transitions

## Abstract

The *SQUAMOSA-PROMOTER BINDING PROTEIN-LIKE* (*SPL*) family of transcription factors is functionally diverse, controlling a number of fundamental aspects of plant growth and development, including vegetative phase change, flowering time, branching, and leaf initiation rate. In natural plant populations, variation in flowering time and shoot architecture have major consequences for fitness. Likewise, in crop species, variation in branching and developmental rate impact biomass and yield. Thus, studies aimed at dissecting how the various functions are partitioned among different *SPL* genes in diverse plant lineages are key to providing insight into the genetic basis of local adaptation and have already garnered attention by crop breeders. Here we use phylogenetic reconstruction to reveal nine major *SPL* gene lineages, each of which is described in terms of function and diversification. To assess evidence for ancestral and derived functions within each *SPL* gene lineage, we use ancestral character state reconstructions. Our analyses suggest an emerging pattern of sub-functionalization, neo-functionalization, and possible convergent evolution following both ancient and recent gene duplication. Based on these analyses we suggest future avenues of research that may prove fruitful for elucidating the importance of *SPL* gene evolution in plant growth and development.

## Introduction

*SQUAMOSA-PROMOTER BINDING PROTEIN-LIKE* (SPL) proteins constitute a diverse family of transcription factors that play fundamental roles in plant growth and development, and are defined by a highly conserved region of 76 amino acids called the SBP domain (Klein et al., 1996; Yang et al., 2007). The SBP domain is involved in both nuclear import and sequence-specific DNA binding to a consensus-binding site containing a GTAC core motif and gene-specific flanking regions (Birkenbihl et al., 2005; Yamasaki et al., 2006; Liang et al., 2008). *SPL* genes are found in all green plants, including single-celled green algae, mosses, gymnosperms, and angiosperms (Cardon et al., 1997; Arazi et al., 2005; Riese et al., 2008), and were first identified in *Antirrhinum majus* (snapdragon, Plantaginaceae, asterid) based on the ability of closely related *AmSBP1* and *AmSBP2* to bind to the promoter of the floral meristem identity gene *SQUAMOSA* (*SQUA*) (Klein et al., 1996). This review focuses on the diversification of *SPL* genes following both gene duplication and speciation events, and illustrates the importance of research into these genes for a better understanding of plant development and evolution.

## Gene Duplication as a Mechanism for *SPL* Gene Diversification

Gene duplication is common in plants and plays a key role in trait evolution (Lawton-Rauh, 2003; Crow and Wagner, 2006; Kaessmann, 2010; Airoldi and Davies, 2012). The *SPL* gene family is an excellent system in which to determine the fate of duplicate genes due to its extensive history of gene doubling, previously identified upstream pathways and downstream targets, and wide range of developmental functions. Whereas the most common fate of gene duplication is functional loss in one copy (non-functionalization), functional evolution can occur through the partitioning of ancestral functions (sub-functionalization), or the acquisition of novel functions (neo-functionalization) in one or both descendent genes (Ohno, 1970; Zhang et al., 1998; Lynch and Conery, 2000; reviewed in Zhang, 2003). Although sub-functionalization may have little immediate impact on phenotype, increased specialization of sub-functionalized paralogs within particular developmental modules is thought to be an important pre-requisite for trait evolution by mitigating the negative effects of mutations in genes that would otherwise exhibit strong pleiotropy (Force et al., 1999; Hughes, 1999; Hittinger and Carroll, 2007). Numerous studies have revealed different fates for duplicate genes in plant development (e.g., Kramer et al., 2004; Causier et al., 2005; Yamaguchi et al., 2006). However, much still remains to be learned about the evolutionary outcome of duplicated genes – both generally and in specific gene lineages – regarding their impact on genetic pathway and trait evolution.

Genomic sequencing has revealed 16, 18, 13, and 31 *SPL* genes in *Arabidopsis thaliana* (*Arabidopsis*, Brassicaceae), *Oryza sativa* (rice, Poaceae), *Physcomitrella patens* (moss, Funariaceae), and *Zea mays* (maize, Poaceae), respectively (Cardon et al., 1999; Arazi et al., 2005; Hultquist and Dorweiler, 2008; Riese et al., 2008; Miura et al., 2010). These genes can be separated into two major groups – long and short – the latter of which are largely regulated by the microRNAs *miR156* and *miR157* (Cardon et al., 1997, 1999; Rhoades et al., 2002; Guo et al., 2008). Although understudied relative to other gene families, functional analyses have revealed divergent developmental roles for *SPL* genes in a diversity of angiosperm taxa. These include the promotion of juvenile to adult phase change (heteroblasty), reproductive transition, trichome development, apical dominance, inflorescence branching, fruit ripening, plastochron length (time between leaf initiation), pollen sac development, and copper homeostasis (Unte et al., 2003; Manning et al., 2006; Wu and Poethig, 2006; Schwarz et al., 2008; Wang et al., 2009; Yamaguchi et al., 2009; Yamasaki et al., 2009; Jiao et al., 2010; Miura et al., 2010; Preston and Hileman, 2010; Yu et al., 2010). Since variation in many of these traits accounts for both inter- and intra-specific variation in lifetime fitness (e.g., Hall and Willis, 2006; Anderson et al., 2011), functional analyses of duplicated *SPL* genes under different environmental conditions may foster substantial insights into the genetic basis for variation in plant life history traits including architecture and phase change evolution.

## Patterns of Duplication in the *SPL* Gene Family

**Figure 1:**
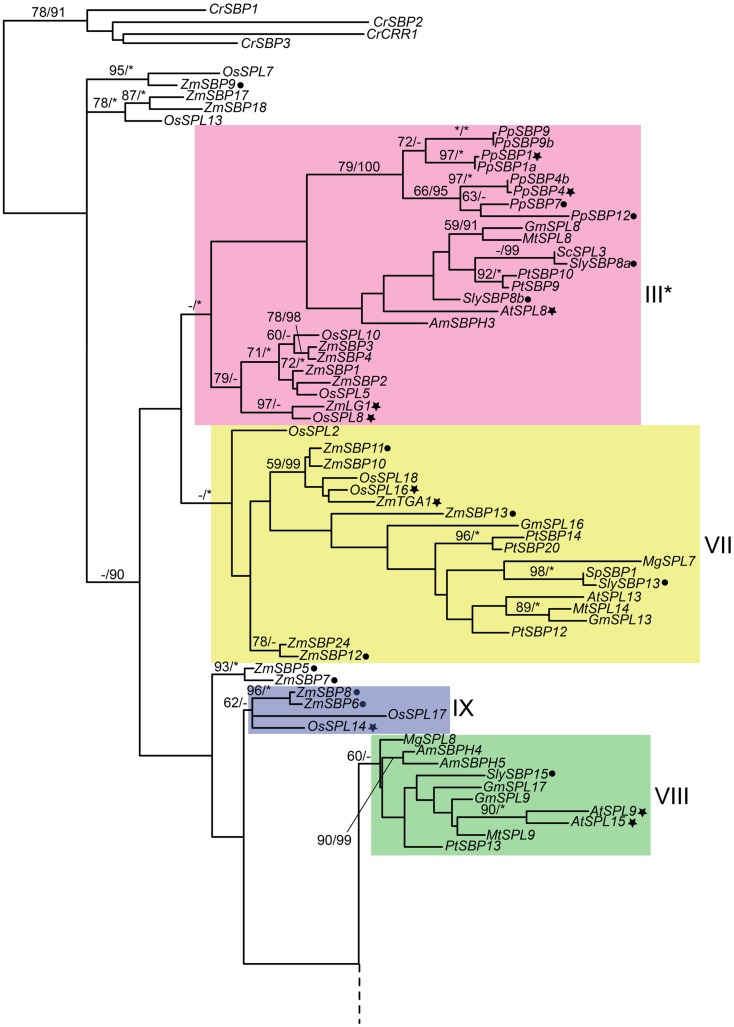

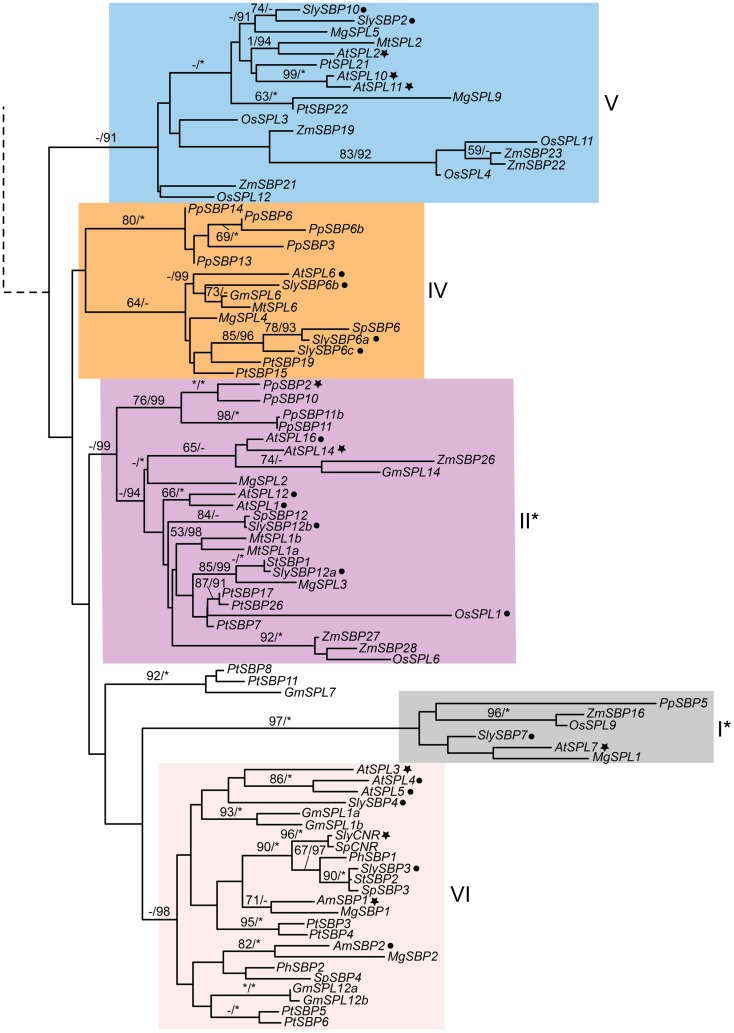
**Phylogeny of *SPL* genes based on an alignment of the SBP-box domain from representative seed plants**. Sequences were downloaded from Genbank/EMBL and Phytozome v8.0 (http://www.phytozome.net/) based on BLAST searches and previously published data (Table S1 in Supplementary Material). Amino acids were aligned by eye in Macclade (Maddison and Maddison, 2003) and nucleotides were subjected to maximum likelihood (ML) in GARLI (Zwickl, 2006) using the best-fitting GTR + I + Γ model of evolution based on results of ModelTest 3.7 (Posada and Crandall, 1998) with 10 random-additions. ML bootstrap values were obtained using 500 bootstrap replicates and are shown below the branches if above 50%; asterisks indicate 100%. Bayesian posterior probability values were obtained in MrBayes 3.2.1. (Ronquist and Huelsenbeck, 2003), with 12 million generations, sampling every 1000th generation, with 25% of trees discarded as burn-in. Values above 90% are indicated to the right of bootstrap values; asterisks indicate 100%. The tree was rooted on genes from the most distantly related species, the green alga *Chlamydomonas reinhardtii*. Genes in clades with asterisks are not regulated by *miRNA*156 or *miRNA*157. Filled star, functionally characterized; filled circle, expression data available. *At*, *Arabidopsis thaliana*; *Am*, *Antirrhinum majus*; *Cr*, *Chlamydomonas reinhardii*; *Gm*, *Glycine max*; *Mg*, *Mimulus guttatus*; *Mt*, *Medicago truncatula*; *Pp*, *Physcomitrella patens*; *Pt*, *Populus trichocarpa*; *Os*, *Oryza sativa*; *Sc*, *Solanum chacoense*; *Sl*, *Solanum lycopersicum*; *Sp*, *Solanum phureja*; *St*, *Solanum tuberosum*; *Zm*, *Zea mays*. Dotted lines connect the upper and lower parts of the tree. Clade colors match Salinas et al. (2012) where applicable.

Similar to the developmentally important MADS-box transcription factor family (Becker and Theissen, 2003), phylogenetic evidence supports retention of multiple *SPL* paralogs following both ancient and more recent duplication events (Yang et al., 2007; Guo et al., 2008; Salinas et al., 2012) (Figure 1). Thus, it is hypothesized that duplicate *SPL* genes have been maintained in the genome by positive Darwinian selection following sub- or neo-functionalization. As will become clear in the following sections, gene orthology in this family does not always predict function. This suggests either common patterns of neo-functionalization or differential sub-functionalization in different *SPL* gene lineages.

The recent availability of several fully sequenced plant genomes has bolstered phylogenetic reconstruction of duplication in the *SPL* gene family. Salinas et al. (2012) recently reported eight (I-VIII) major *SPL* clades, at least four of which predate the diversification of embryophytes (land plants) based on a neighbor-joining algorithm, whereas Guo et al. (2008) identified six major clades. To independently determine support for these different *SPL* gene clades, we analyzed a slightly different dataset of *SPL* genes derived from sequences available in Genbank and Phytozome version 9.0 (Table S1 in Supplementary Material) under the GTR + I + γ model of evolution in a maximum likelihood (ML) framework. The model of evolution was selected according to results of MrModelTest version 3.7 (Posada and Crandall, 1998), and ML analyses were run in GARLI with 500 bootstrap replicates (Zwickl, 2006). Bayesian posterior probabilities were also obtained in MrBayes version 3.2.1. (Ronquist and Huelsenbeck, 2003) with 12 million generations, sampling every 1000th generation, and discarding 25% of trees as burn-in. Results of these analyses support the eight major clades described in Salinas et al. (2012), and suggest a possible ninth clade containing the domestication gene *OsSPL14* from rice (Figure 1). Since surveys of available genomic data suggest that *SPL* genes are absent from fungi and metazoans, the ML tree is rooted with genes from the green alga *Chlamydomonas reinhardtii*, which are each other’s closest relatives in unrooted trees. Posterior probability values support the monophyly of clades I, II, III, V, VI, and VII (Figure 1). However, despite some support within clades, there is low support for relationships among major lineages (Figure 1), probably due to the limited number of informative characters within the alignable SBP-box domain (Salinas et al., 2012).

**Figure 2:**
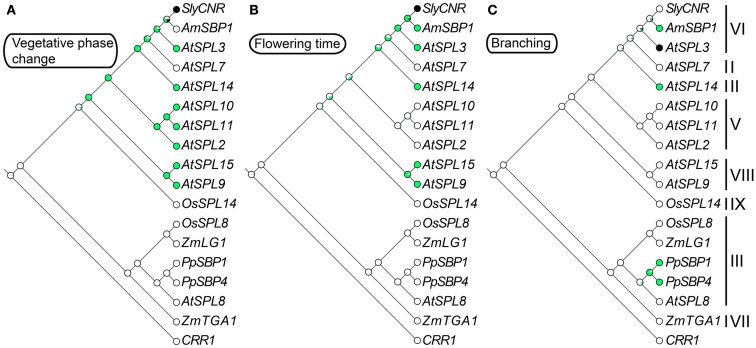
**Ancestral character state reconstructions across the *SPL* gene family**. The gene trees are based on Figure 1, but only include genes for which functional data are available. Character state transitions in vegetative phase change **(A)**, flowering time **(B)**, and branching **(C)** were inferred from a ML analysis in Mesquite version 2.5 (Maddison and Maddison, 2008) under the Mk1 (single rate of change between states) model of evolution. Circles denote the probability of having (green) or not having (white) a particular function. Black circles are coded as unknown.

Intron numbers are highly variable in the gene family, ranging from 1 to 10 (Cardon et al., 1999; Kropat et al., 2005; Guo et al., 2008; Salinas et al., 2012). However, evidence based on available genomic sequences support conservation of exon-intron structures within clades, with the exception of clade-II and clade-IV (Figure 1) (Cardon et al., 1999; Guo et al., 2008; Salinas et al., 2012). In the following sections we summarize our knowledge on the expression and function of representative genes for each clade given available data. In order to better understand the relative timing and number of origins of *SPL* gene functions, the ancestral state for several traits was reconstructed using a ML framework in Mesquite version 2.01 based on pruned versions of the Figure 1 tree (Maddison and Maddison, 2003, 2008) (Figure 2). Only traits with both presence (coded 1) and absence (coded 0) data were included in the analysis. We discuss these results in the context of the importance of different *SPL* clades on green plant evolution and development, and suggest important directions for future research.

## *SPL* Gene Clade Evolution

### Clade-I

*SPL* clade-I genes are characterized by their large size, lack of *miRNA156* and *miR157*-binding sites, and near ubiquitous expression across different organs of the plant (Cardon et al., 1999; Wang et al., 2010; Salinas et al., 2012). For example, in *Solanum lycopersicum* (tomato) *SlySBP7* is expressed in roots, leaves, stems, shoot apices, flowers, and fruits at early to late stages of development (Salinas et al., 2012). Functional analysis within clade-I is limited to *AtSPL7* from *Arabidopsis* and supports a role for this gene in the regulation of copper homeostasis (Yamasaki et al., 2009). Copper is an essential micronutrient required for healthy plant growth and development. However, whereas low levels of copper can result in stunted growth and reduced reproductive output, high levels can be toxic. Thus, maintaining a specific copper concentration in cells has major benefits for plant fitness (Clemens et al., 2001; Puig et al., 2007).

Under low copper conditions *atspl7* mutants have much lower levels of the microRNAs *miR397a*, *miR398b*, *miR398c*, *miR408*, and *miR857*, which in the wild type collectively and negatively regulate copper homeostasis proteins (Abdel-Ghany and Pilon, 2008; Yamasaki et al., 2009). In the case of *miR398b* and *miR398c*, decreased expression in the *atspl7* mutant is due to the loss of *AtSPL7* binding to the Cu-response element (CuRE), characterized by the core sequence GTAC, in the promoter region (Yamasaki et al., 2009). In turn, low levels of *miR398b* and*miR398c* result in the loss of Cu/Zn SUPEROXIDE DISMUTASE 1 (CSD1) and CSD2 degradation, which is normally essential for the reallocation of limited copper supply to support photosynthetic functioning (Sunkar et al., 2006; Yamasaki et al., 2007, 2009; Abdel-Ghany and Pilon, 2008).

**Figure 3:**
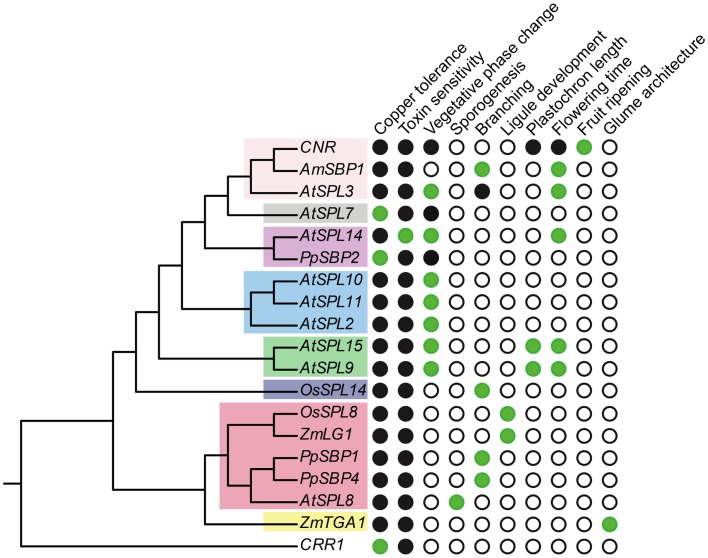
**Functional diversity of *SPL* genes**. The gene tree is based on Figure 1, but only includes genes for which functional data are available. Green circles, presence; white circles, absence; black circles, unknown.

Other *SPL* genes that function as copper-responsive gene promoter binding proteins are *COPPER RESPONSE REGULATOR 1* (*CRR1*) in *Chlyamydomonas reinhardtii* (green alga) and *PpSBP2* in *P. patens* (clade-II) (Kropat et al., 2005; Nagae et al., 2008; Castruita et al., 2011; Strenkert et al., 2011). Similar to *AtSPL7*, *CRR1* binds to GTAC motifs of CuREs, and in *C. reinhardtii* this results in the transcriptional activation of copper-deficiency target genes (e.g., *CYTOCHROMEC6* and *CPX1*) that cause a physiological shift to copper-independent photosynthesis (Quinn and Merchant, 1995; Quinn et al., 1999). However, unlike *AtSPL7*, *CRR1*, and *PpSBP2* are not members of *SPL* clade-I (Figure 1). This suggests either that a function in copper homeostasis has evolved multiple times independently, or has been lost multiple times following speciation and gene duplication (Figure 3). Given the lack of data for this trait across a diversity of *SPL* genes, future studies explicitly testing for copper homeostasis in divergent SPL protein clades will be required to distinguish between these alternative hypotheses.

### Clade-II

Similar to clade-I genes, members of *SPL* clade-II are relatively large, are expressed widely across the plant and throughout ontogeny, and lack negative regulation by *miR156* and *miR157* (Cardon et al., 1999; Stone et al., 2005; Xie et al., 2006; Chuck et al., 2007; Yang et al., 2007; Wang et al., 2010; Salinas et al., 2012). *Arabidopsis* has four clade-II genes – *AtSPL1*, *AtSPL12*, *AtSPL14*, and *AtSPL16* – derived from two recent and one ancient duplication event(s) (Yang et al., 2007) (Figure 1). Expressed sequence tag (EST) and microarray data (eFP Browser: http://bar.utoronto.ca/efp/cgi-bin/efpWeb.cgi) suggest that all four paralogs are expressed widely in seedlings, rosette, and cauline leaves, shoot apical meristems, flowers, fruits, and roots (Winter et al., 2007; Yang et al., 2007). However, whereas *AtSPL16* is expressed most strongly in mid stage shoot apices and cauline leaves, *AtSPL1*, *AtSPL12*, and *AtSPL14* are expressed most strongly in cauline leaves, flowers, and late stage shoot apices (Winter et al., 2007). In tomato, the clade-II genes *SlySBP12a* and *SlySBP12b* are expressed ubiquitously and constitutively from seedling to ripe fruit (Salinas et al., 2012); a similar expression pattern has been described for *OsSPL1* in rice (*Oryza sativa*) (Yang et al., 2007).

Functional data exists for two clade-II genes, *AtSPL14* and *PpSBP2*; these data suggest functional diversification following speciation in this clade (Stone et al., 2005; Nagae et al., 2008). Similar to its distant paralogs, *CRR1* (outgroup) and *AtSPL7* (clade-I), *PpSBP2* is involved in the regulation of copper homeostasis in *P. patens* (Nagae et al., 2008) (Figure 1). Under high copper conditions abundant PpSBP2 binds to GTACT motifs in the promoter of *IRON SUPEROXIDE DISMUTASE* (*FeSOD*), resulting in its transcriptional repression (Nagae et al., 2008). However, since SODs are critical for eliminating the harmful effects of reactive oxygen species (ROS), produced as by-products of cellular metabolism, ROS accumulation is mitigated by the switch to copper/zinc SOD catalysis (Mittler, 2002). By contrast, when copper and zinc are limiting, it is hypothesized that *PpSBP2* expression is low, resulting in the reinstatement of *FeSOD* functioning.

In the case of *arabidopsis*, mutations in *AtSPL14* result in plants that fail to respond to the fungal toxin fumonisin B1 (FB1) (Stone et al., 2005). In wild type plants, FB1 exposure causes apoptosis, presumably as a defense mechanism against its negative effect on sphingolipid metabolism (Desai et al., 2002). By contrast, growth of *atspl14* mutants is not inhibited on media containing FB1 (Stone et al., 2005). In addition to FB1 resistance, *atspl14* mutants are defective in early developmental phase change (Stone et al., 2005) (Figure 3). In early development wild type *arabidopsis* plants undergo a physiological transition from juvenile to adult growth. This phase change is accompanied by subtle changes in leaf morphology, and results in vegetative adult plants that are competent to respond to floral inductive signals (Poethig, 1990; Telfer et al., 1997; Baürle and Dean, 2006). Reduction-of-function *atspl14* mutants have a developmentally faster transition to the adult growth phase relative to wild type plants, which slightly accelerates flowering time (Stone et al., 2005). Thus, it is inferred that *AtSPL14* functions to delay the juvenile to adult transition. This result is in striking contrast with other characterized *arabidopsis* *SPL* genes in clades V, VI, and VIII (see sections 8, 9, and 11) that function to accelerate vegetative phase change (Figures 2A and 3). How *SPL* paralogs evolved antagonistic effects on developmental phase change awaits further molecular genetic inquiry.

### Clade-III

Members of clade-III *SPL* genes have diverse functions, and although short relative to clade-I and II genes, lack regulation by *miR156* or *miR157* (Cardon et al., 1999; Xie et al., 2006; Salinas et al., 2012). The clade-III gene *AtSPL8* was the first *SPL* gene to be functionally characterized in *arabidopsis* (Unte et al., 2003). Mutations in *AtSPL8* have no effect on phase change, but have a profound effect on seed set, petal trichome production, and root growth (Unte et al., 2003; Zhang et al., 2007). This is modulated through the positive (flowers) and negative (roots) regulation of gibberellic acid (GA) signaling (Zhang et al., 2007). At late stages of flower development stamen filaments of *atspl8* mutants are shorter than wild type and anthers produce fewer pollen grains due to a reduction in sporogenous cells undergoing meiosis; a similar, but weaker, reduction in meiosis is also observed in the ovule (Unte et al., 2003). As a consequence, seed set in *atspl8* mutants is strongly reduced (Unte et al., 2003; reviewed in Xing et al., 2011). Interestingly, this phenotype is amplified in quadruple *spl8:spl2:spl9:spl15* mutants, although some pollen viability remains (Xing et al., 2010). Thus, since *AtSPL2*, *AtSPL9*, and *AtSPL15* are members of clades V, VIII, and VIII, respectively, these data support either functional conservation or independent recruitment of *SPL* genes in microsporogenesis.

Despite the lack of functional data, expression of the *AtSPL8* co-orthologs *SlySBP8a* and *SlySBP8b* in tomato tentatively suggests at least partial conservation of function in megasporogenesis. Both genes are expressed more highly in carpels and young versus old fruits, but have very low expression in roots, seedlings, and stamens (Salinas et al., 2012). By contrast, expression of the *AtSPL8* ortholog *VvSBP19* in grapevine (*Vitis vinifera*) has not been detected in fruits (Wang et al., 2010). The moss *P. patens* has between five and eight clade-III *SPL* genes, *PpSBP1/1b*, *PpSBP4/4b*, *PpSBP7*, *PpSBP9/9b*, and *PpSBP12*, two of which have been functionally characterized. Loss-of-function *ppsbp1* and *ppsbp4* mutants produce more branches than wild type at both early and late stages of colony development, and are defective in spore germination (Riese et al., 2008) (Figure 2C). Furthermore, *PpSBP1*, *PpSBP4*, *PpSBP7*, and *PpSBP12* are negatively regulated by cryptochromes, blue-light absorbing photoreceptors involved in regulating phase change and the circadian clock (Guo et al., 1998; Somers et al., 1998; Devlin and Kay, 2000; Riese et al., 2008). Although functionally distinct, *AtSPL8* expression is also regulated by cryptochromes. However, this regulation is positive rather than negative. It is postulated that this difference in regulation is the result of differential dominance of the gametophytic and sporophytic stages of mosses and land plants, respectively (Folta et al., 2003; Riese et al., 2008).

In maize and rice, the *SPL* clade-III gene *liguleless1* (*Zmlg1*and *OsSPL8*) is involved in development of the ligule and auricle (Figure 3), two structures borne on the adaxial surface of grass leaves between the blade and the sheath (Moreno et al., 1997; Lee et al., 2007). In *lg1* mutants, the ligule and auricle are completely missing due to the loss of longitudinal periclinal divisions, and the blade-sheath boundary is less well defined than wild type (Sylvester et al., 1990). Interestingly, grasses vary greatly in whether they possess ligules and/or auricles (Lee et al., 2007). Thus, it is hypothesized that changes in the expression or function of *lg1*-like genes explain variation for these leaf traits. This hypothesis waits further testing.

### Clade-IV

Members of clade-IV *SPL* genes include *AtSPL6* from *arabidopsis*, *SlySBP6a*, *SlySBP6b*, and *SlySBP6c* from tomato, and *PpSBP3*, *PpSBP6*, *PpSBP6b*, *PpSBP13*, and *PpSBP14* from *Physcomitrella* (Figure 1). No orthologs have been found in monocots suggesting a loss of this gene lineage at least in the fully sequenced genomes of rice and other grasses. The only *SPL* clade-IV gene to be functionally characterized is *P. patens PpSBP3* (Cho et al., 2012). Deletion of *PpSBP3* accelerates and increases the number of gametophore-producing leafy buds in the moss gametophyte, suggesting that it normally represses reproductive development (Cho et al., 2012). Although not directly comparable due to the lifecycle differences of mosses and angiosperms, this function is somewhat similar to *AtSPL14* (clade-II), which functions to delay the transition to adult development (Stone et al., 2005).

Sequence analyses and expression data in *miR156* and *miR157* mutants suggest that clade-IV *SPL* genes are regulated by both *miR156* and *miR157* (Cho et al., 2012; Salinas et al., 2012). In tomato expression of the three clade-IV *SPL* genes suggest divergence of function. With the exception of stems, *SlySBP6a* is expressed constitutively across the plant, similar to *AtSPL6* in *arabidopsis* (Cardon et al., 1999; Salinas et al., 2012). By contrast, *SlySBP6c* expression is confined to shoot apical meristems and the whole inflorescence, but is not expressed in vegetative tissues, flowers, or fruits. Finally, *SlySBP6b* expression increases during fruit development (Salinas et al., 2012). It will be interesting to functionally test the role of *SlySBP6b* in fruit maturation – a key agronomic trait – and to determine whether the differential gene expression of these tomato genes reflect sub- or neo-functionalization.

### Clade-V

In contrast to *SPL* genes that affect both the timing of and morphological features associated with phase change (see sections 9 and 11), silencing of the closely related *SPL* clade-V paralogs *AtSPL10*, *AtSPL11*, and *AtSPL2* only affects the latter (Figure 2A) (Shikata et al., 2009). During vegetative growth, the first rosette leaves of plants with reduced *AtSPL10*, *AtSPL11*, and *AtSPL2* expression are narrower, more oval, and have more serrated edges than their wild type counterparts, characteristic of later developing rosette leaves. Furthermore, following the onset of inflorescence development, the late developing cauline leaves of *AtSPL10*, *AtSPL11*, and *AtSPL2* silenced plants are wider compared to late developing leaves on wild type plants, and have adaxial trichomes that are normally only found on early cauline leaves (Shikata et al., 2009). Taken together, these data show that growth and development during the vegetative and reproductive phases of the *arabidopsis* life cycle can be uncoupled.

In the case of *AtSPL10*, *AtSPL11*, and *AtSPL2* silenced plants changes in leaf characteristics are likely the result of reduced *FRUITFULL* (*FUL)* expression, which has long been known to affect leaf development (Gu et al., 1998; Shikata et al., 2009). However, unlike *ful* mutants, mutations in *AtSPL10*, *AtSPL11*, and *AtSPL2* have no effect on inflorescence or fruit development (Shikata et al., 2009). This suggests that *FUL* is differentially regulated in leaves, inflorescence meristems and fruits. It will be important to determine how this regulation is partitioned among *arabidopsis* *SPL* genes, and to see if this differential regulation is conserved in other species, including the other *miR156*-regulated clade-V rice genes *OsSPL3*, *OsSPL4*, *OsSPL11*, and *OsSPL12* (Xie et al., 2006), and the constitutively expressed tomato paralogs *SlySBP2* and *SlySBP10* (Salinas et al., 2012) (Figure 1).

In addition to leaf morphology, *AtSPL10* and *AtSPL11* have been implicated in cell differentiation during early embryogenesis (Nodine and Bartel, 2010). *Dicer-like 1* (*dcl1*) mutants have decreased miRNA expression, resulting in derepression of several hundred miRNA target genes, including *AtSPL10* and *AtSPL11*, and precocious differentiation of early embryonic cells. However, when *dcl1* mutants are crossed with *atspl10:atspl11* mutants, normal embryogenesis is partially restored (Nodine and Bartel, 2010). These data demonstrate redundant roles for *AtSPL10* and *AtSPL11* in both early and late stage differentiation.

### Clade-VI

The most widespread reported function of *SPL* genes is promoting the transition from juvenile to adult growth (Figure 3), which is marked by an increase in responsiveness to floral inductive signals, resulting in competence to flower (Baürle and Dean, 2006). In *arabidopsis*, phase change occurs primarily in response to environmental signals, such as temperature and developmental age, and is accompanied by subtle changes in leaf morphology (Poethig, 1990; Telfer et al., 1997). Overexpression analyses implicate *AtSPL3*, and tentatively the other two clade-VI genes *AtSPL4* and *AtSPL5*, in the timing of and/or morphological features associated with phase change (Wu and Poethig, 2006). These functions are likely moderated through negative regulation by *miR156* (Wu and Poethig, 2006; Schwarz et al., 2008; Shikata et al., 2009; Wang et al., 2009; Yamaguchi et al., 2009).

Constitutive expression of *miR156*, which results in a decrease in expression of 10 out of 16 *arabidopsis* *SPL* genes including *AtSPL3/4/5*, results in prolongation of the vegetative phase, as well as delayed flowering, and an increase in the number of juvenile leaves (Schwab et al., 2005; Wu and Poethig, 2006). In mutants that overexpress the clade-VI genes *AtSPL3*, *AtSPL4*, and *AtSPL5*, and the clade-VIII gene *AtSPL15* (see Section 11), leaves develop adult characteristics – including abaxial trichomes, and an increase number of cells that are smaller in size – faster than their wild type counterparts (Wu and Poethig, 2006; Usami et al., 2009). This is in contrast to the overexpression of the clade-II gene *AtSPL14*, which results in a truncated juvenile vegetative phase (Stone et al., 2005). Based on these incomplete data, ancestral trait reconstructions suggest that a role in vegetative phase change evolved fairly early during the diversification of angiosperm *SPL* genes, and was followed by multiple losses of function outside *arabidopsis* (Figure 2A). However, with mutants available for only nine out of 16 *arabidopsis* *SPL* genes, and very few characterized *SPL* genes from other species, the exact timing of this evolutionary transition is equivocal (Figure 2A).

**Figure 4:**
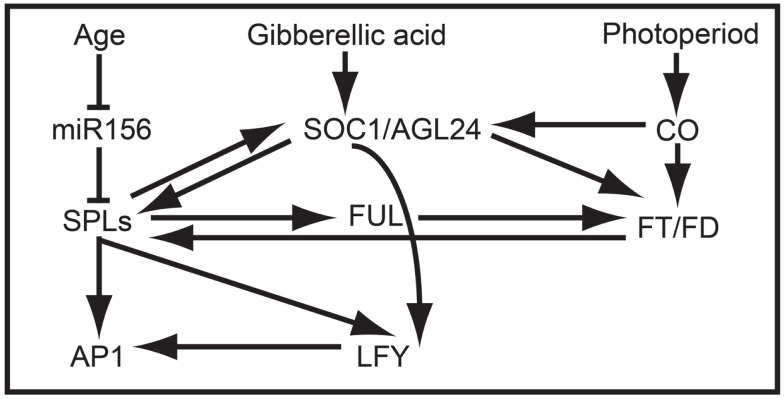
***SPL* clade-VI gene-dependent flowering network in *Arabidopsis***. The at least partially redundant genes *AtSPL3*, *AtSPL4*, and *AtSPL5* are negatively regulated by *miR156* in early development and positively regulated by *SOC1* prior to flowering. In turn, *AtSPL3*, *AtSPL4*, and *AtSPL5* induce the expression of *FUL*, *AP1*, *LFY*, and *SOC1*, resulting in the production of flowers.

In addition to promoting vegetative phase change, overexpression data suggest that *AtSPL3*, *AtSPL4*, and *AtSPL5* redundantly promote the reproductive transition by integrating signals from the autonomous, photoperiod, age, and GA pathways (Figures 2B and 4) (Cardon et al., 1997; Gandikota et al., 2007; Wang et al., 2009; Yamaguchi et al., 2009; Jung et al., 2012; Porri et al., 2012; Yu et al., 2012). Under short day conditions, all three *SPL* genes are negatively regulated in an age dependent manner by *miR156*, and are positively regulated by SUPPRESSION OF OVEREXPRESSION OF CONSTANS1 (SOC1) through the GA pathway (Jung et al., 2011, 2012). By contrast, under long day conditions, SOC1, FLOWERING LOCUS T (FT), and FLOWERING LOCUS D (FD) positively regulate *AtSPL3*, *AtSPL4*, and *AtSPL5* in leaves in response to photoperiod signals (Jung et al., 2012). SPL proteins indirectly activate *FT* expression, probably through the direct binding of the inflorescence meristem gene *FUL*, and directly activate transcription of *FUL*, *APETALA1 (AP1)*, and *LEAFY* (*LFY*) in the shoot apical meristem (Corbesier and Coupland, 2006; Corbesier et al., 2007; Wang et al., 2009; Yamaguchi et al., 2009) (Figure 4).

Functional data exist for a few core eudicot clade-VI genes, allowing preliminary comparative analysis following speciation. Similar to *arabidopsis*, the single *AtSPL3/4/5* ortholog *AmSBP1* in snapdragon is involved in initiating flower development within the inflorescence. However, in contrast to *arabidopsis* plants constitutively expressing *miR156*, where flower production is delayed, silencing of *AmSBP1* can eliminate flowering completely (Preston and Hileman, 2010). One hypothesis for these different phenotypes in *arabidopsis* and snapdragon is a tighter control of *AmSBP1* versus *AtSPL3/4/5* on the expression of downstream floral organ identity genes, including the *AP1/FUL*-like genes *SQUA*, *DEFH28*, and *AmFUL*, and the *LFY*-like gene *FLORICAULA* (*FLO*) (Klein et al., 1996; Preston and Hileman, 2010). Alternatively, the less extreme phenotype associated with simultaneously silencing *AtSPL3*, *AtSPL4*, *AtSPL5*, and several other *arabidopsis* *SPL* genes, versus silencing of a single snapdragon *SPL* gene, might be explained by antagonistic functions of *arabidopsis* miRNA-regulated *SPL* genes. To test this antagonism hypothesis, loss-of-function phenotypes must be generated and compared for all *arabidopsis* *SPL* genes, starting with the clade-VI genes *AtSPL4* and *AtSPL5*.

In addition to the flowering phenotype, silencing of *AmSBP1* also causes an increase in vegetative branching under long days (Figure 2C) (Preston and Hileman, 2010). This branched phenotype is similar to that observed under short day growth conditions, suggesting that it is a consequence of late flowering rather than a loss of apical dominance *per se* (Preston and Hileman, pers. obs.). By contrast in tomato, mutations in the *AtSPL3/4/5* ortholog *COLORLESS NON-RIPENING* (*CNR*) result in fruits that fail to ripen (Manning et al., 2006). At present, this can be ascribed as a novel *SPL* gene function (Figure 3). Indeed, differential expression of the closest *CNR* homologs, *SlySBP3*, and *SlySBP4*, suggest that these genes are not involved in fruit ripening, but may function in earlier carpel development (Salinas et al., 2012). It will be interesting to see if tomato *SPL* genes other than *CNR* have been recruited to function in fruit ripening, as suggested by gene expression data (Salinas et al., 2012), and if paralogous *SPL* genes affect fruit development in related species with (e.g., *Solanum melongena*, eggplant, Solanaceae) or without (e.g., *Petunia x hybrida*, petunia, Solanaceae) fleshy fruits (Pabón-Mora and Litt, 2011).

In addition to the lineage containing *AtSPL3*, *AtSPL4*, and *AtSPL5*, another clade-VI gene lineage exists for which there is no *arabidopsis* ortholog (Figure 1). Based on data from snapdragon, this clade of genes also targets expression of *AP1/FUL*-, *FT*-, and *LFY*-like genes (Klein et al., 1996; Preston and Hileman, 2010). Future characterization of genes within this will potentially allow more accurate reconstruction of the ancestral functions of clade-VI genes and promises to bolster our understanding of developmental differences between rosid and asterid core eudicots.

### Clade-VII

Expression and functional data in *arabidopsis*, maize, and rice implicate clade-VII genes in various aspects of above ground plant development. In *arabidopsis*, the single *SPL* clade-VII gene *AtSPL13* has been implicated in delaying leaf outgrowth following emergence of the cotyledons during germination (Martin et al., 2010a,b). Loss of negative regulation of *AtSPL13* due to mutations in the *miR156* binding site significantly delays emergence of leaf primordia probably due to the concomitant upregulation of *miR157*, which normally represses *AP2*-like genes such *SCHNARCHZAPFEN* (*SNZ*) (Martin et al., 2010a,b). In addition to hypocotyls, *AtSPL13* is broadly expressed in different plant organs (Martin et al., 2010b). Thus, it will be interesting to determine if *AtSPL13* has other functions in plant development, such as those described for clade-VII *SPL* genes below.

The most extensively characterized clade-VII gene in maize is *TEOSINTE GLUME ARCHITECTURE 1* (*TGA1*), variation in which explains the drastic difference in fruitcase morphology between cultivated maize and its ancestor teosinte (*Zea mays* ssp. *parviglumis*) (Wang et al., 2005). Whereas teosinte has hard fruitcases resulting from invagination of the inflorescence branch and hardening of the floral bract (glume), maize has a soft fruitcase that allows easy harvesting of the fruit. Evidence strongly suggests that the difference in fruitcase morphology between subspecies is due to a single amino acid substitution in *TGA1* (Wang et al., 2005; Preston et al., 2012). However, amino acid and gene/protein expression analyses suggest that the parallel evolution of soft fruitcases in other grasses is under the regulation of other, as yet unknown, genes (Preston et al., 2012). Furthermore, the actual function of *TGA1* in teosinte is still under investigation.

Despite the lack of correlation between *TGA1* structure and expression, research on one of two *TGA1* homologs in rice suggests that clade-IX *SPL* genes function generally in late reproductive development (Wang et al., 2012). Specifically, in a recent study it was demonstrated that increased grain size and grain number in the rice *indica* variety HJX74 compared to the rice *indica* Basmati varieties are positively correlated with *OsSPL16* expression (Wang et al., 2012). These differences are mediated by changes in patterns of cell proliferation and elongation, resulting in associated changes in grain shape. Unlike *SPL* genes in many other clades, silencing of *OsSPL16* has no affect on phase change or plant architecture. However, constitutive expression in *arabidopsis* and rice accelerates flowering and results in dwarf plants with fewer inflorescence branches, respectively (Wang et al., 2012). Together these data suggest that the regulatory interactions between many SPL proteins and their downstream targets (e.g., *AP1/FUL-*, *LFY-*, *FT*-, and *SOC1*-like genes) may be largely conserved (Figure 4), but that differential expression is driving broad-scale functional differences.

In addition to *TGA1*, maize has five other clade-VII genes, one of which has an expression profile consistent with its playing a role in feminization (Hultquist and Dorweiler, 2008) (Figure 1). Maize plants are monecious, developing a terminal male inflorescence (tassel) and lateral female inflorescences (ears). Tassels and ears differ substantially in terms of their developmental timing, branching architecture, flower and reproductive organ abortion, and floral bract (glume) morphology (Kiesselbach, 1999). In the *tasselseed1* (*ts1*) and *mediator of paramutation1* (*mop1*) mutants, tassels are feminized relative to wild type tassels (Dorweiler et al., 2000; Acosta et al., 2009). Interestingly, *ZmSBP11* is expressed more highly in these mutant feminized tassels than in wild type tassels, suggesting a role in one or all of the ear-specific traits (Hultquist and Dorweiler, 2008). It will be of great interest to discern if and how *ZmSBP11* affects ear development and to see whether similar functions can be assigned to the *AtSPL13* homologs in *arabidopsis*, constitutively expressed *SlySBP13* homolog in tomato, and the *OsSPL2*, and *OsSPL18* homologs in rice (Salinas et al., 2012) (Figure 1).

### Clade-VIII

Redundant roles in vegetative phase change and reproductive transition have been demonstrated for the closely related *miR156*-regulated clade-VIII genes *AtSPL9* and *AtSPL15* (Schwarz et al., 2008; Usami et al., 2009). Mutations in these genes cause a subtle increase in vegetative rosette leaves, but have no significant effect on inflorescence cauline leaves (Schwarz et al., 2008). However, in the double *spl9 spl15* mutant, vegetative rosette leaf number is significantly increased relative to the single mutants, leaves are more rounded, and flowering time is delayed relative to wild type (Schwarz et al., 2008). Furthermore, overexpression of *AtSPL9* in *hyponastic leaves1* (*hyl1)* mutants that have lowered *miR156* expression, and therefore higher *SPL* gene expression, results in the complete loss of the juvenile phase (Li et al., 2012). Ancestral state reconstructions suggest that vegetative phase change and flowering time function evolved in the *SPL* gene lineage prior to diversification of the core eudicots, but the exact timing of these transitions is ambiguous (Figures 2A,B). Thus, future work is needed to determine whether the shared function of the *arabidopsis* *SPL* clade-V, VI, and VIII genes is due to ancient neo-functionalization, followed by multiple losses, or alternatively, multiple independent evolutionary gains.

In addition to phase change, plastochron length is affected in late flowering *atspl9atspl15* double mutants, suggesting dissociation between growth and development (Figure 3) (Schwarz et al., 2008). To date, this is the only clade of genes for which a role in plastochron length has been described (Figure 3). The *atspl9atspl15* double mutant has a shortened plastochron relative to wild type, which is correlated with a smaller shoot apical meristem and rounded leaves (Schwarz et al., 2008). However, in the *more and smaller cells 1d* (*msc1d*) mutant that overexpresses *AtSPL15* due to disruption of the *miR156* binding site, the juvenile to adult phase transition is accelerated (Figure 2A). This acceleration is accompanied by an increased ratio of rosette leaves having adult characteristics – adaxial trichomes and more smaller cells – similar to overexpression lines of *AtSPL3*, *AtSPL4*, and *AtSPL5* (Wu and Poethig, 2006; Usami et al., 2009). Thus, leaf number and identity is correlated with phase change, but it can be uncoupled.

Recent genetic evidence also suggests that *AtSPL9* is involved in petal trichome initiation, through the activation of *TRICHOMELESS* (*TCL1*), and anthocyanin pigment accumulation in vegetative stems (Yu et al., 2010; Guo et al., 2011). In the case of pigment production, overexpression of *AtSPL9* results in reduced levels of anthocyanin, suggesting a negatively regulatory interaction in wild type plants. The mechanism of this negative regulation is hypothesized to be interference of the MYB-bHLH-WD40 complex, which controls the transcription of flavonoid biosynthesis genes (Guo et al., 2011). It is as yet unknown whether a role in trichome development and pigment production is specific to *AtSPL9*, or whether *SPL* homologs have similar functions in other species.

### Clade-IX

In maize, the clade-IX genes *ZmSBP6* and *ZmSBP8*, and the currently unplaced paralogs *ZmSBP5* and *ZmSBP7* (Figure 1), are hypothesized to be involved in feminization (Hultquist and Dorweiler, 2008) (Figure 1). Evidence supporting this comes from gene expression analyses in *ts1* and *mop1* mutants, where transcript levels of both genes are higher in feminized versus non-feminized tassels (Hultquist and Dorweiler, 2008). However, since this correlation in gene expression does not imply causation, future functional studies are needed to test this hypothesis.

Convincing evidence suggesting a role for *SPL* clade-IX grass genes in branching, which has apparently evolved multiple times independently during *SPL* gene diversification (Figure 2C), comes from genetic work on the rice domestication gene *OsSPL14*, which is strongly associated with the *WEALTHY FARMER’S PANICLE* QTL for variation in rice architecture (Jiao et al., 2010; Miura et al., 2010). Increased expression of *OsSPL14* in the *japonica* line Shaoniejing relative to the *indica* line Taichung Native 1, and ST-12 relative to *japonica* Nipponbare, results in decreased vegetative branching and increased inflorescence branching, the latter resulting in an increase in grain number (Jiao et al., 2010; Miura et al., 2010). It remains unclear how *OsSPL14* is able to at once both promote and repress branching in different developmental contexts (i.e., vegetative versus inflorescence). However, expression of *OsSPL14* is negatively regulated by *OsmiR156* (Miura et al., 2010).

The opposite action of *OsSPL14* on vegetative and inflorescence branching has major implications for breeding. In the case of rice, increased expression of this gene is favorable as it increases grain yield at the expense of biomass. However, in forage and biofuel grasses increased biomass is favorable to grain yield. Indeed, Fu et al. (2012) recently demonstrated that by differentially increasing the expression of *PvmiR156* in switch grass (*Panicum virgatum*), some plants had good characteristics for biofuel engineering, including increased leaf number, late flowering, increased vegetative branch number, and reduced inflorescence size. This phenotype resulted from the differential targeting of eight *SPL* genes. Thus, research aimed at elucidating the specific function of different *SPL* genes in crop species and their relatives has great promise for fine-tuning plant architecture in an agricultural context.

## Future Directions and Concluding Remarks

Accepting the caveat that deep branches in the most likely *SPL* trees are not well supported (Figure 1), phylogenetic state reconstructions based on functionally characterized *SPL* genes from a few distantly related taxa tentatively suggest the origin of early and late phase change function at least prior to the diversification of core eudicots (Figures 2A,B). However, if the ancestor of clades I, II, V, VI, and VIII had a dual role in regulating vegetative and reproductive transitioning, this function has subsequently been partitioned through differential sub-functionalization following both gene duplication and speciation (Figures 2A,B). Furthermore, there is evidence of both neo-functionalization (e.g., in fruit ripening and glume architecture) and parallel recruitment (e.g., in branching) in several different lineages (Figures 2C and 3).

The inferred dynamic history of *SPL* gene function across both gene clades and species might be explained by a combination of changes in regulation, biochemical function, and/or downstream targeting. Indeed, it is already known that most, but not all, *SPL* genes are regulated by microRNAs in an age dependent manner, and that there have been shifts in regulation between the cryptochrome, photoperiod, and GA pathways (Cardon et al., 1997; Guo et al., 1998; Somers et al., 1998; Devlin and Kay, 2000; Gandikota et al., 2007; Zhang et al., 2007; Riese et al., 2008; Wang et al., 2009; Yamaguchi et al., 2009; Jung et al., 2012; Porri et al., 2012). Furthermore, sequence analyses and comparisons of gene expression in *SPL*-silenced versus wild type plants suggest both conservation and divergence of *SPL* protein targets. The most intriguing case is the regulatory relationship between SPL proteins and the meristem identity *AP1/FUL*-like genes. Sequence and expression analyses suggest that either these regulatory relationships evolved early and have subsequently been lost multiple times following the divergence of the *TGA1*-like clade from other angiosperm *SPL* clades, or that these regulatory relationships have evolved multiple times independently (Klein et al., 1996; Shikata et al., 2009; Preston and Hileman, 2010). Testing these alterative hypotheses across multiple *SPL* clades and species will be important to determine the mechanistic basis for functional changes within the gene family.

In addition to the mechanistic basis of changes in function, characterization of multiple *SPL* genes from different taxa has generated hypotheses regarding the diversification of key ecological traits, such as the evolution of growth habit. Annual and perennial growth habits can be distinguished on the basis of quantitative differences in flowering time, meristem dormancy/identity, architecture, biomass, and/or woodiness, all of which are affected in *spl* and their target *ap1/ful*-like gene mutants (Melzer et al., 2008). Thus, *SPL* genes are good candidates underlying shifts between annuity and perenniality, possibly through the differential targeting of genes involved in meristem identity and phase change. A promising avenue of research will be to study related annual and perennial populations or species to see if growth habit differences can be explained by differences in expression and/or protein functional differences of microRNA-regulated *SPL* genes. Such studies are currently underway for different populations of *Mimulus guttatus* and different species of *Arabidopsis* that vary in several traits related to growth habit.

## Conflict of Interest Statement

The authors declare that the research was conducted in the absence of any commercial or financial relationships that could be construed as a potential conflict of interest.

## Supplementary Material

The Supplementary Material for this article can be found online at http://www.frontiersin.org/Plant_Evolution_and_Development/10.3389/fpls.2013.00080/abstract
